# Agitation-dependent biomechanical forces modulate GPVI receptor expression and platelet adhesion capacity during storage

**DOI:** 10.1186/s12959-021-00359-7

**Published:** 2022-01-12

**Authors:** Ehteramolsadat Hosseini, Amin Solouki, Masood Haghshenas, Mehran Ghasemzadeh, Simone M. Schoenwaelder

**Affiliations:** 1 Blood Transfusion Research Centre, High Institute for Research and Education in Transfusion Medicine, Tehran, Iran; 2 Charles Perkins Centre, The University of Sydney, Camperdown, NSW Australia; 3 Heart Research Institute, Newtown, NSW Australia

**Keywords:** Agitation, Glycoprotein VI (GPVI), Platelet, Shedding, Shear stress, Src-kinases, Thrombosis, Transfusion

## Abstract

**Background:**

Continuous agitation during storage slows down the platelet storage lesions. However, in special circumstances, manual-mixing can be alternatively used to store products for short time periods without compromising platelet quality. Based on this finding, and given the role of shear stress in modulating receptor expression, we were interested in comparing the levels of platelet adhesion receptor, GPVI and platelet adhesion capacity under each storage condition.

**Methods:**

Platelet concentrates (PCs) were divided into three groups: continuously-agitated PCs (CAG-PCs) with or without PP2 (Src kinase inhibitor) and manually-mixed PCs (MM-PCs). Platelet count/MPV, swirling, GPVI and P-selectin expression, GPVI shedding, platelet adhesion/spreading to collagen were examined during 5 days of storage.

**Results:**

While MM- and CAG-PCs showed similar levels of P-selectin expression, GPVI expression was significantly elevated in MM-PCs with lower GPVI shedding/expression ratios, enhanced platelet adhesion/spreading and swirling in manually-mixed PCs. Of note, CAG-PCs treated with PP2 also demonstrated lower P-selectin expression and GPVI shedding, higher GPVI expression and attenuated swirling and spreading capability.

**Conclusion:**

Given the comparable platelet activation state in MM and CAG-PCs as indicated by P-selectin expression, enhanced platelet adhesion/spreading in MM-PCs, along with relatively higher GPVI expression here, supports previous studies demonstrating a role for biomechanical forces in modulating GPVI-dependent function. Thus, lower GPVI expression in CAG-PCs may be due to shear forces induced by agitation, which keeps this receptor down-regulated while also attenuating platelet adhesion/spreading capacities during storage. Low platelet function in PP2-CAG-PCs also highlights the importance of Src-kinases threshold activity in maintaining platelets quality.

**Supplementary Information:**

The online version contains supplementary material available at 10.1186/s12959-021-00359-7.

## Research highlights

- In the absence of agitator, manual mixing can maintain platelet quality for up to 4 days of storage
- Both manually-mixed (MM) and agitated PCs had a comparable platelet activation state for 4 days
- MM-PCs showed higher levels of GPVI expression with lower GPVI shedding/expression ratios
- MM-PCs showed enhanced platelet adhesion/spreading and swirling during storage
- lower platelet counts and MPV in CAG-PCs may be due to agitation-dependent microvesiculation
- Lower GPVI expression and platelet adhesion capacity in CAG-PCs may be due agitation-dependent biomechanical forces

## Background

According to existing blood banking guidelines following their preparation, platelet concentrates (PCs) are stored within a temperature range of 20 to 24 °C, under continuous agitation. This is a basic protocol to maintain maximum platelet function as well as prevent unwanted platelet activity during storage [1, 2]. However, while it may be possible to maintain temperature within the specified range, the ability to constantly agitate platelet products in all situations can be challenging. There are several reasons for interrupting continuous agitation, which in addition to platelet transport can include the lack of a suitable agitator/incubator in small blood transfusion centers or unequipped hospital blood banks and pre-transfusion delay of platelet products in hospital wards [3]. These describe some of the frequent challenges faced by hospitals and transfusion centers especially in developing countries. Therefore, studies that can determine the maximum allowable interruption in agitation of PCs are considered to be of significant importance to many transfusion researchers thus far. Some of these studies have shown that even under static conditions, platelets can maintain their optimal function with minimal changes for up to 24 h provided that after this period of agitation pause, they immediately return to the appropriate conditions [4–7]. However beyond these studies, some researchers are also looking for scientific methods that can maintain the efficiency and function of platelets while further prolonging the agitation break time [3–6]. One method is to use manual mixing of platelet products, a technique first introduced by Mitchell et al. at West Midlands Blood Transfusion Centre, United Kingdom [8]. In this innovative method, while observing the standard storage temperature, instead of continuous agitation, the researchers used manual mixing of PCs in different directions for 30 s every 24 h. They then demonstrated that in a four-day period of platelet storage, no significant differences were observed between continuously-agitated (CAG) PCs and manually-mixed (MM) ones with respect to metabolic conditions, functional response in aggregation tests, release of platelet granular contents including ATP and Beta-thromboglobulin (β-TG), and post-transfusion recovery. These promising findings suggested manual platelet mixing as an appropriate emergency strategy for platelet storage.

Given the findings that long-term storage of platelets under static conditions intensifies platelet storage lesion (PSL) [6, 8], studies demonstrating preserved platelet quality via manual-mixing versus continuous agitation strategy may also provide an opportunity to explore the unique effects of agitation on platelet function. While the importance of biomechanical forces in modulating platelet activation and behaviour is accepted [9–11], the effect of agitation or biomechanical collision forces on platelet function and viability during storage have not been well characterized yet. Several lines of evidence indicate that agitation or biomechanical collision forces can impact some platelet in vitro markers, while even diminishing in vivo platelet post-transfusion recovery under some specific conditions [1, 12–14]. These conclusions were derived from studies where the application of different agitation techniques were applied, including increasing agitation (rpm), or comparing flat-bed, circular and elliptical agitators that induce different levels of mechanical shear stress in platelet concentrates during storage. We therefore re-evaluated some of the qualitative indicators of platelet storage using the manual mixing method, and further extended these studies to compare other important platelet activity indices, including the expression levels of P-selectin, GPVI shedding and its expression as well as platelet adhesion to collagen using the CAG and MM-storage conditions. We were particularly interested to examine GPVI expression, because if the levels of platelet activity are similar in the MM- and CAG-PCs, any differences in expression levels and/or shedding of this receptor may be due to agitation per se, given previous studies indicated that shear stress may enhance GPVI shedding [15–17]. Finally, the study presented here also evaluated platelet adhesion/ spreading capacity, in order to highlight the functional significance of any differences in the expression of this adhesion receptor incurred under different storage conditions.

## Methods and materials

### Reagents and chemicals

For reagents and chemicals, see Additional file 1.

### Sample preparation

20 PRP-PCs units were obtained from eligible, volunteer donors after obtaining informed consent. All products passed release procedures under Iranian Blood Transfusion Organization (IBTO) standard protocols. Each bag contained at least 1 × 10^9^ platelets/mL in ~ 70 mL of autologous plasma based on the criteria of Association for the Advancement of Blood & Biotherapies (AABB). Following preparation (Day 0), two ABO- and D- matched PRP-PC units were pooled in a closed system using a connecting device instrument (TSCD-II, Terumo Sterile Tubing Welder, Japan). The combined bag (~ 140 mL) was manually mixed and then split using a similar connection device into three independent platelet bags from same company, ensuring identical volumes for each (still met the standard volume) with the use of a digital scale. The three bags were labeled i) GAC-PC (PC which kept under continuous agitation), ii) PP2-CAG-PC (CAG-PC treated with Src kinase inhibitor PP2) and iii) MM-PC (PC stored with manual mixing, in the absence of agitation, according to described method by Mitchell et al. [8]. Under sterile condition, the Src kinase inhibitor PP2 dissolved in buffer was added to the allocated bag (to a final concentration of 10 μM), while the same buffer without inhibitor (vehicle) was also added to either GAC-PC or MM-PC labelled bags. All bags were then stored at 20–24 °C in their designated agitation conditions. Samples (2 mL of product) were collected from each bag cord under sterile conditions for the required analyses on days 1, 3, 4 and 5 of storage. From each sample, washed platelets were isolated and re-suspended in Tyrode’s buffer as described previously [18] . For flow cytometry and adhesion analysis, platelet count was adjusted to 2 × 10^7^ /ml. Platelet-poor plasma (PPP), obtained from PRP with the platelet count of 5 × 10^8^ /mL were also subjected to two steps ultracentrifugation (2 × 10^4^ g for 30 min each time) and MP-free supernatants were separated and kept in − 20 °C to be analyzed by western blot (WB) or ELISA [19]. The study was approved by the local Ethics committee, and informed consent was obtained from all blood donors, according to IBTO protocol.

### Flow cytometry to determine GPVI and P-selectin surface expression

Platelets were stained with either fluorochrome-conjugated anti CD62P (P-selectin) or GPVI. Samples were subjected to flow cytometer (CyFlowSpace, Partec GmbH, Germany) to quantify the expressions of P-selectin and GPVI (see Additional file 1 for further details).

### Analysis of GPVI shedding

GPVI shedding was analysed using 2 difference procedures: i) To detect soluble GPVI, microparticle-free PPP was subjected to an in-house ELISA assay. For full description of method see Additional file 1. ii) To validate our ELISA results, microparticle-free PPP was also subjected to SDS-PAGE and western blotting analysis. Albumin was fractionated from PPP samples prior to each experiment, to avoid any interference of proteins bands due to large amounts of albumin in plasma [20]. (see Additional file 1 for further details).

### Static platelet adhesion to a collagen matrix

Adhesion and spreading of fluorochrome labeled platelets on collagen-coated coverslips were visualized using fluorescence microscopy (100x objective), and total number of adherent platelets, as well as percentage (%) of spread platelets was calculated. Briefly in this method visualized adhered platelets were divided into 3 patterns, simply adhered platelets with no obvious protrusions, platelet with filopodial or lamellipodial formations, and platelet with extensive circumferential lamellipodial formations extended to full spreading of cell around its central core. As already described [21], the percentage of these fully spread platelets to other adhered platelets was quantified during platelet storage. (see Additional file 1 for further details).

### Swirling assessment of PCs

Swirling assessment is a simple and useful macroscopic method to evaluate PC quality on a large scale. Discoid platelets when viewed against bright background refract the light in such a way that by moving the PC manually, the expert technician can see the glow of tiny particles flowing in the direction of motion, which is so-called the “swirling” phenomenon. Lack of swirling is associated with lactate accumulation and a decrease in pH in PCs, while platelets also lose their discoid shape and functional capacities under this condition [22, 23]. Transfusion of PCs lacking swirling is also thought to poorly increase the number of platelets (reduced Corrected Count Increment) while also raising the risk of a blood transfusion reaction [24]. In our study swirling scores are graded as: Score 3 (excellent), very clear homogeneous swirling observed in all part of the bag; Score 2 (good), clear homogeneous swirling observed in all part of the bag; Score 1 (poor), partial swirling only observed in some part of the bag which is not clear in general; Score 0 (no swirling), homogeneous turbidity with no swirling which is not changed with pressure or movement [22].

### QC parameters of PCs

See Additional file 1.

### Statistical analysis

See Additional file 1.

## Results

### Significant reduction in P-selectin expression in PP2 (Src inhibitor) treated PCs during storage

**Fig. 1:**
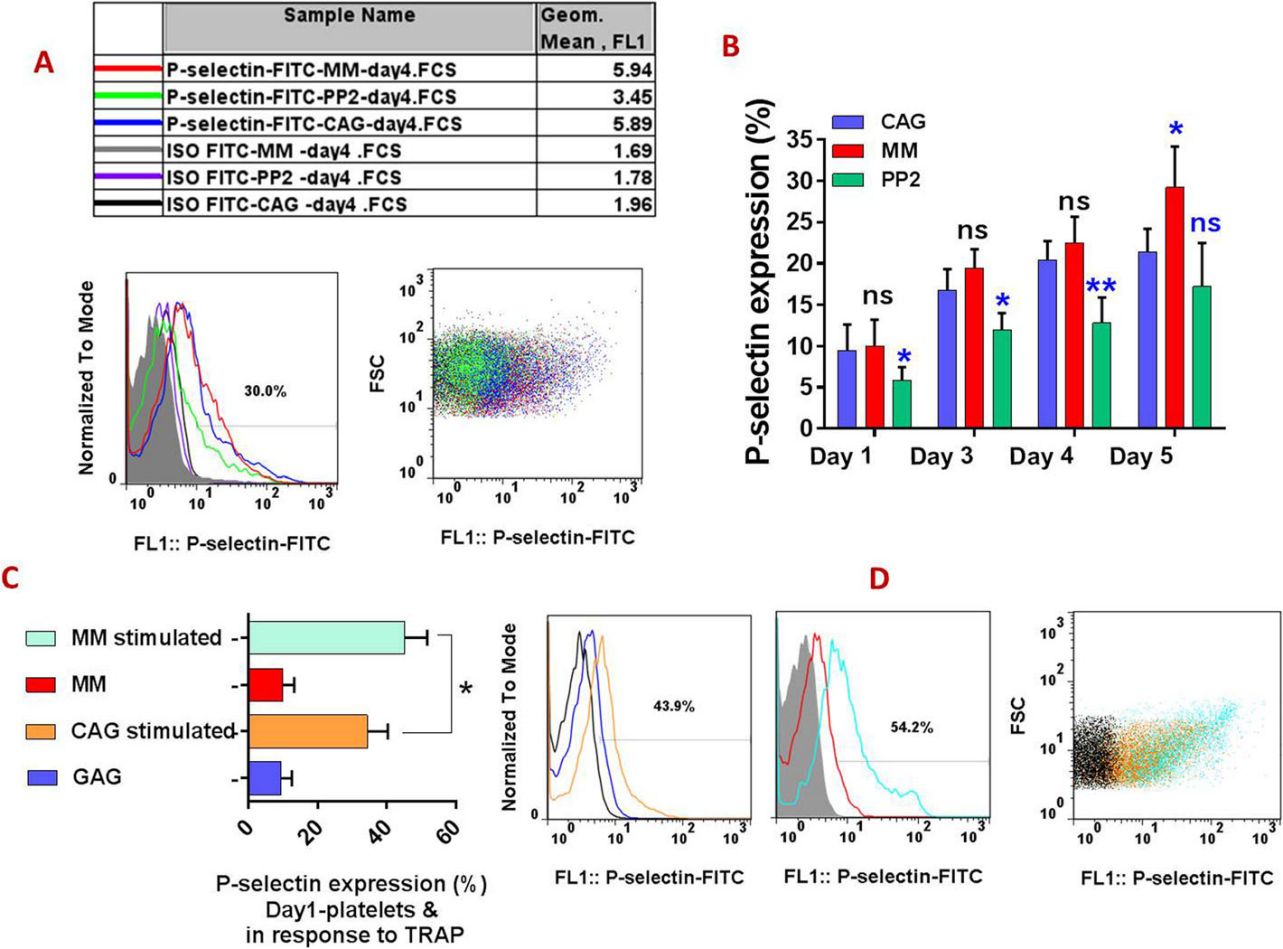
P-selectin expression in manually mixed and agitated PCs during storage. The representative dotted plot and corresponding histogram show 4-day stored platelets P-selectin levels in both MM-PCs and CAG-PCs as well as CAG-PCs in presence of PP2 (**A**). The graph **B** compares P-selectin expression in mentioned groups of PCs during 5 days of storage (*n* = 10). Graph **C** shows higher P-selectin expression in response to TRAP (5 μM) in one-day stored MM-PCs compared to CAG-ones. This is also shown by representative dotted plot and corresponding histogram (**D**). CAG = continuously-agitated; MM = manually-mixed;.PC = platelet concentrate. Note: ns: not significant; *p < 0.05

As shown in Fig. 1A & B, treatment of CAG-PC with the Src kinase inhibitor PP2 (10 μM) resulted in a significant reduction in P-selectin expression in platelets stored until the fifth day (compared with CAG-PC in the presence of vehicle alone). While a decrease was observed at day 5, this was not significant. The level of P-selectin expression presented in this figure has been presented as the percentage of positively gated platelets, while a representative FACS profile in Fig. 1A also shows the levels of P-selectin expression in 4-day stored PCs based on geometric mean fluorescence intensity.

### Comparable levels of P-selectin expression in manually mixed versus continuously- agitated PCs during 4 days of storage

During the first 4 days of storage, a comparable level of P-selectin expression was demonstrated in MM- and CAG-PCs. In contrast, P-selection expression was significantly higher on day 5 of storage in MM-PCs. Interestingly, despite comparable levels of P-selection expression on the initial day of storage, agonist-induced P-selectin exposure, an indicator of granule release and platelet activation in response to 5 μM TRAP was significantly higher in one-day stored MM platelets versus those of CAG-PCs (Fig. 1C), with expression level of P-selectin in MM platelets reaching 54% in response to TRAP stimulation, whereas this increase in CAG platelets was up to 42%.

### The patterns of GPVI expression in response to different agonists

**Fig. 2:**
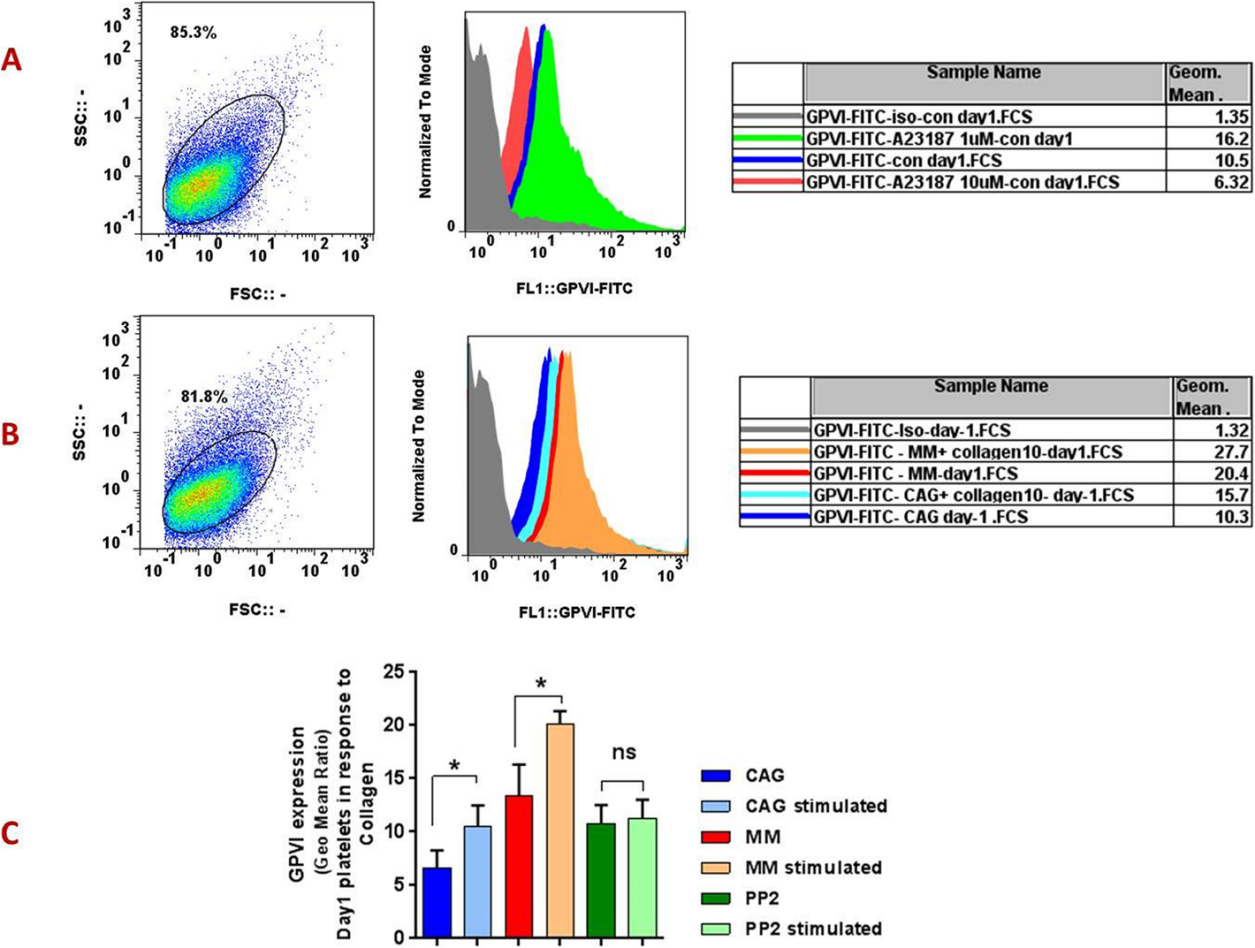
GPVI expression in response to different agonists. The representative histograms show different levels of GPVI expression in response to low (1 μM) and high (10 μM) concentrations of potent agonist ionophore A23187 (**A**) as well as 10 μg/ml collagen (**B**) in one-day stored CAG platelets. Graph (**C**) also depicts the significant increments of GPVI in response to collagen in both MM- and GAC- PCs whereas this agonist did not increase GPVI expression in PP2 treated GAG-PCs (all in one-day stored PCs, *n* = 5). CAG = continuously-agitated; MM = manually-mixed;.PC = platelet concentrate. Note: ns: not significant; *p < 0.05

As shown in Fig. 2A the treatment of one-day stored platelet with 1 μM ionophore A23187 increased GPVI expression by more than 1.5-fold whereas increasing the ionophore concentration to 10 μM reduced the GPVI level by more than 1.6 times less. The histogram shown in Fig. 2B also exhibited an increase in GPVI expression in response to collagen (10 μg / ml) for both one-day stored MM- and GAG-PCs. However, while these induced increases in GPVI were significant for both mentioned products, collagen stimulation did not significantly alter GPVI expression in CAG-PCs treated with PP2 (Fig. 2C).

### Reduced GPVI shedding concomitant with increased expression levels in PP-2 treated CAG-PCs during storage

**Fig. 3:**
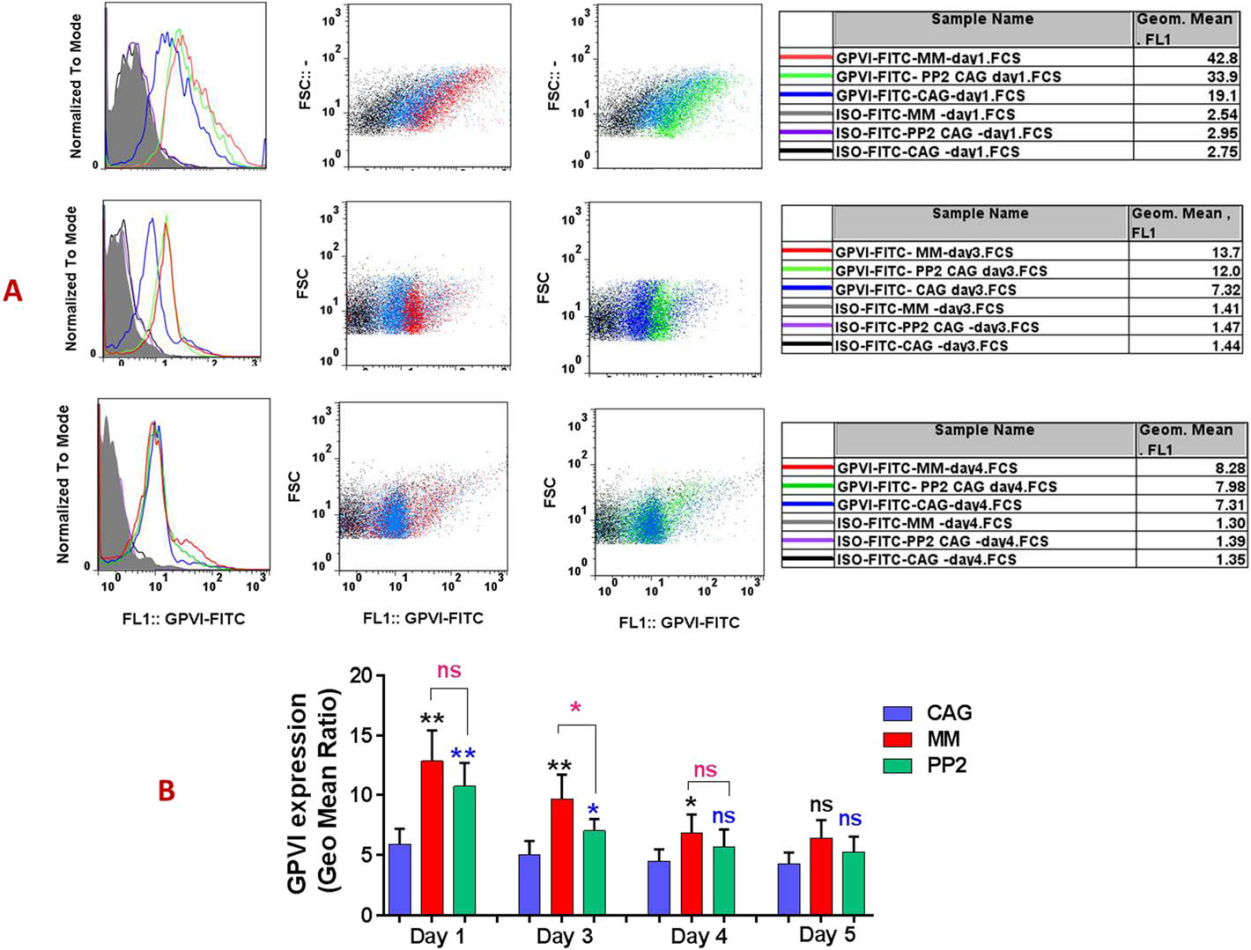
GPVI expression in manually mixed and agitated PCs during storage. The representative dotted plots and corresponding histograms (**A**) shows higher levels of GPVI expression in both MM-PCs and PP2-treated CAG-PCs compared to non-treated CAG-PCs during 4 days of storage (in one,3 and 4-day stored PC). Graph **B** compares the levels of GPVI expression among mentioned groups of PCs (*n* = 10). CAG = continuously-agitated; MM = manually-mixed; .PC = platelet concentrate. Note: ns: not significant; *p < 0.05; **p < 0.01

**Fig. 4:**
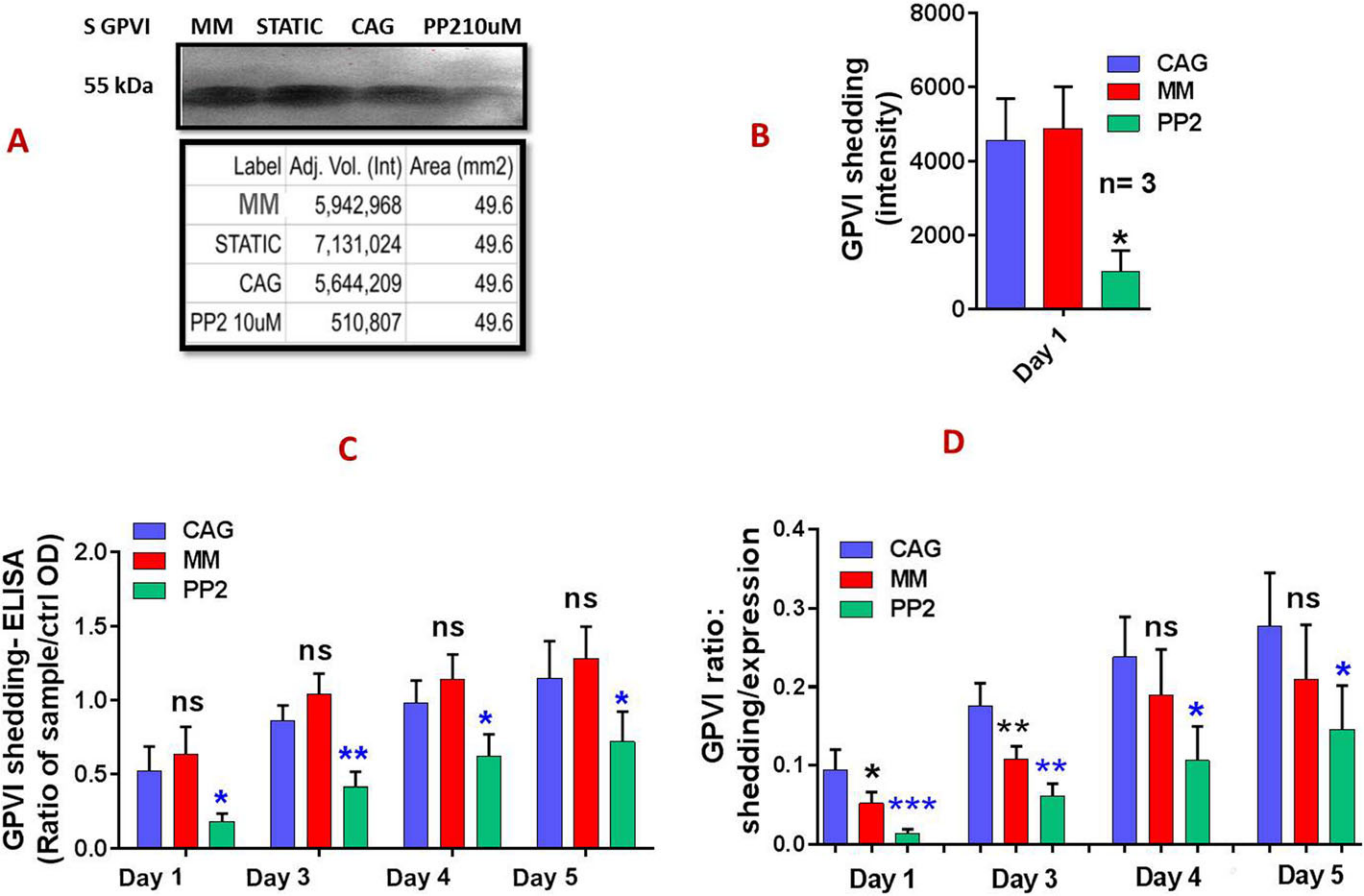
GPVI shedding in manually mixed and agitated PCs during storage. **A** demonstrates representative western blot image illustrating shedding patterns of GPVI in one-day stored MM-PCs and CAG-PCs (in presence and absence of PP2 treatment). Graph **B** also compares GPVI shedding of those mentioned products (*n* = 3). Graph **C** compares different levels of GPVI shedding, detected by ELISA method among MM-PCs, CAG-PCs and PP2-treated CAG-PCs (n = 10) during storage. To better demonstrate significant differences between MM-PCs and CAG-PCs, the ratio of GPVI shedding to GPVI expression for each product were calculated and compared (**D**). CAG = continuously-agitated; MM = manually-mixed; .PC = platelet concentrate. Note: ns: not significant; *p < 0.05; **p < 0.01, ***p < 0.001

Inhibition of Src kinase with PP2 treatment significantly increased GPVI expression on stored platelets compared to CAG-PCs until the fourth day of storage. While expression of this receptor on days 4 and 5 of storage was somewhat lower, these decreases were not statistically significant (Fig. 3 A&B). Consistent with this and as shown in Fig. 4, GPVI shedding was significantly inhibited by PP2. Figure 4 A&B depicts western blot analysis of platelet supernatants from PP2 treated and untreated platelets, where GPVI shedding was evaluated in one-day stored platelets. As shown here, Src inhibitors significantly suppressed GPVI shedding to the lowest level. Similar results were also obtained using ELISA analysis, where the levels of GPVI shedding were significantly lower in the presence of PP2 during storage (Fig. 4C). The demonstration of the ratio of shedding to total receptor expression during the storage period also shows a significant reduction of these values in the treated versus non-treated platelets while both (either PP2 treated CAG-PCs or non-treated CAG-PCs) were similarly under agitation (Fig. 4D).

### GPVI expression/ectodomain shedding in manually mixed and agitated PCs during storage

**Fig. 5:**
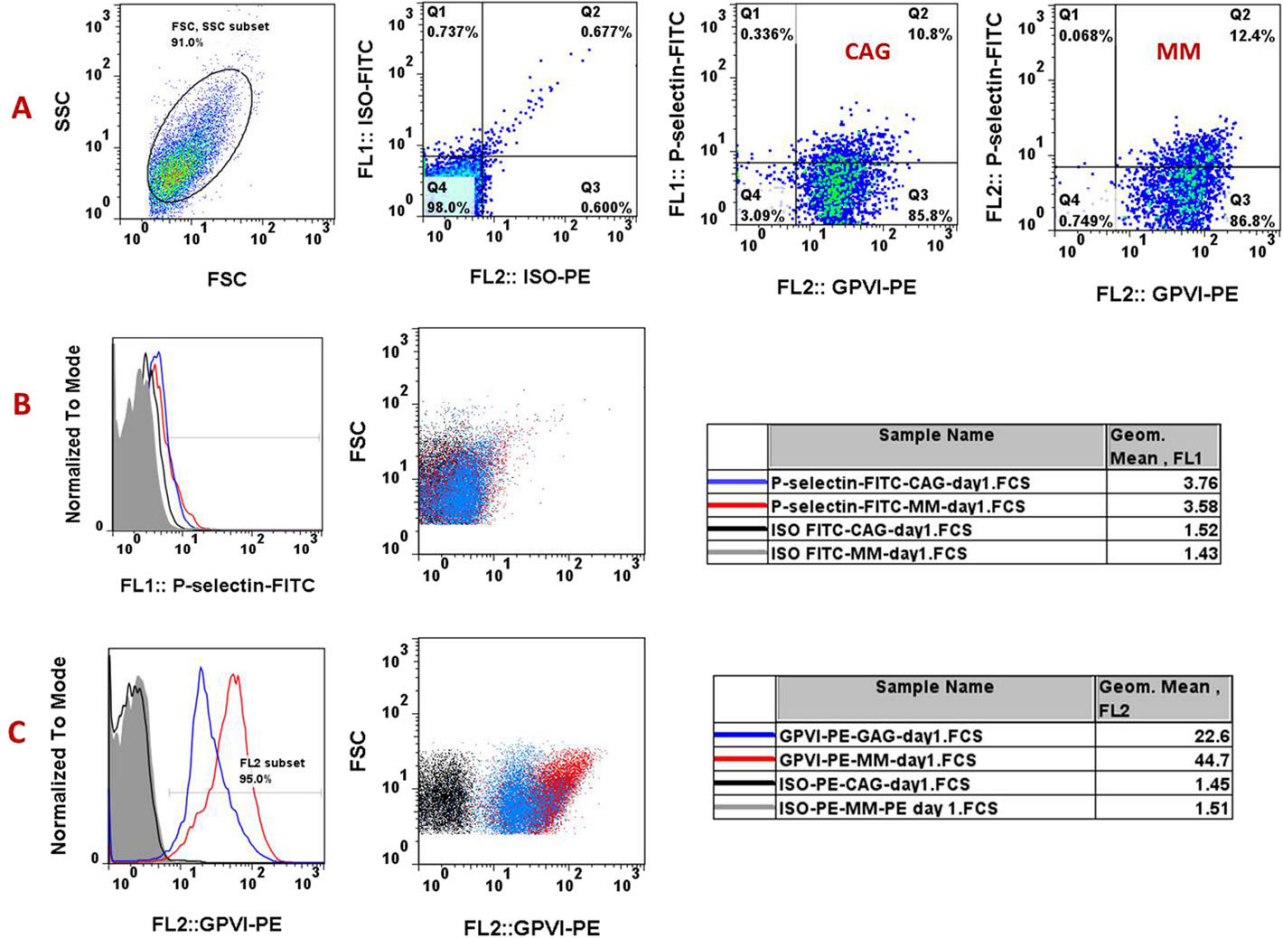
Dual staining of GPVI expression and P-selectin in one-day stored manually mixed and agitated PCs. In order to confirm the previous findings that indicate different levels of GPVI in MM- and CAG-PCs with the same levels of P-selectin in these products, simultaneous staining of these receptors was performed one-day stored PCs. **A** shows a representative dotted plots of double stained platelets with FITC-anti P-selectin and PE-anti GPVI in either MM-PC or CAG-PCs (one-day stored PCs). **B** and **C** demonstrate representative histograms and their corresponding dotted plot for each of P-selectin and GPVI expression separately. (n = 10); CAG = continuously-agitated; MM = manually-mixed; .PC = platelet concentrate

As shown in Fig. 3A & B, GPVI expression levels were improved in MM platelets compared to those stored under CAG conditions. These results demonstrated significantly higher levels of GPVI expression in MM-PCs during storage, with the exception of day 5, where expression was still higher than CAG-PCs while there was no significant increase. However, in contrast to that observed in PCs treated with the Src kinase inhibitor, this increase in expression was not accompanied by a decrease in the levels of GPVI shedding in MM-PCs or an increase in those under continuous agitation. As presented in Fig. 4 B, western blot analysis did not show any significant differences between the GPVI shedding levels of MM- and GAG-PCs. This was confirmed through assessment of PCs using ELISA, which also demonstrated no difference in the rate of GPVI shedding between the two groups of MM- and CAG-PCs during storage (Fig. 4C). However, as shown in Fig. 4D, the evaluation of the shedding to total expression ratio shows a higher value in CAG platelets, at least in platelets stored for less than 4 days. Finally, due to the higher expression of GPVI in MM-PCs compared to CAG-PC, which was associated with comparable levels of P-selectin in these two groups, one-day stored platelets from each group were subjected to dual staining with PE- GPVI and FITC-P-selectin antibodies. These studies further confirmed our previous observations, with evidence of higher levels of GPVI expression in MM-PCs while their P-selectin levels were comparable with those of CAG-PCs (Fig. 5A, B&C).

### Prevention of platelet spreading on a collagen matrix in the presence of Src inhibitors during storage

**Fig. 6:**
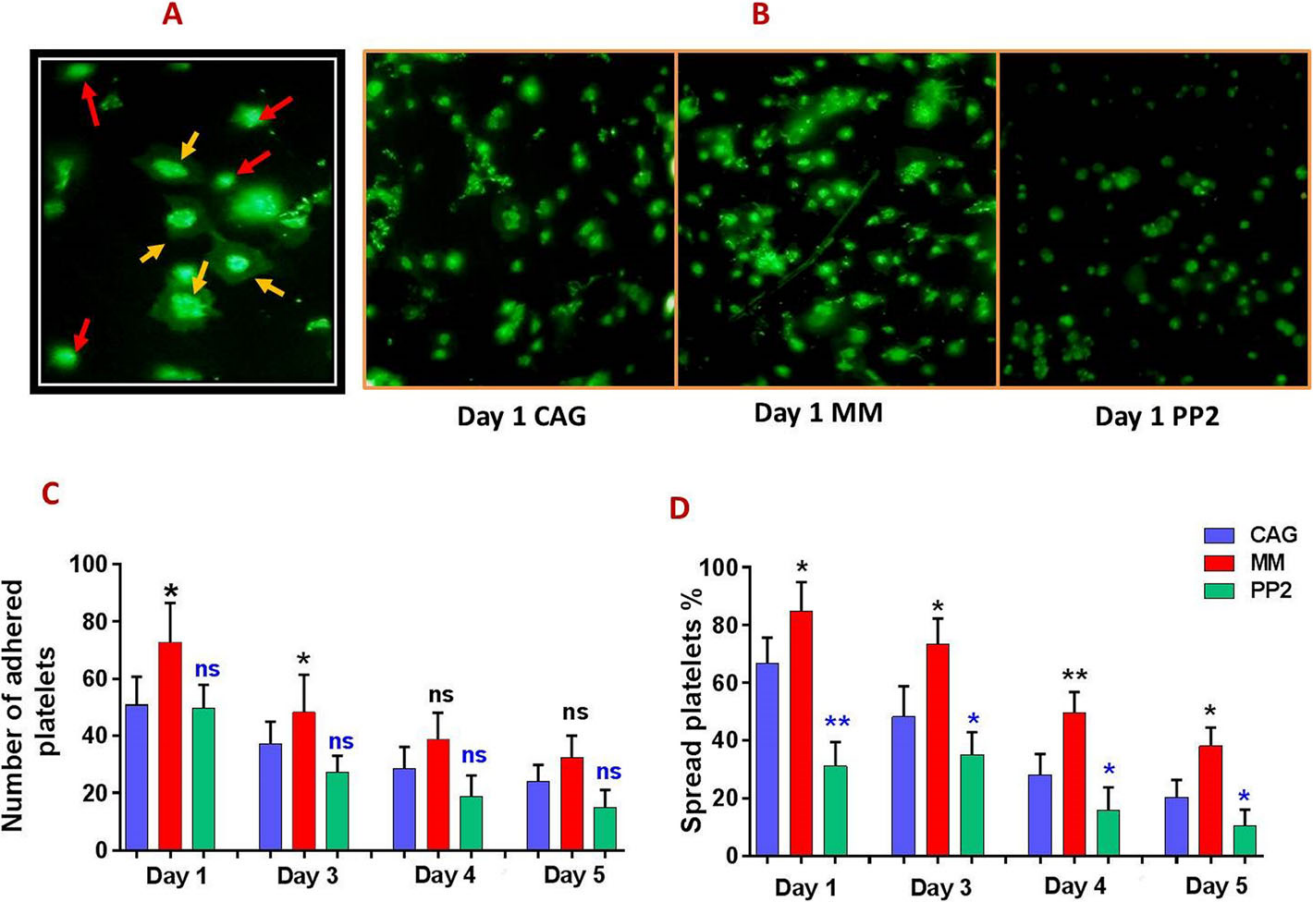
Platelet adhesion and spreading over collagen matrix in manually mixed and agitated PCs during storage. **A** provides a demonstrative image characterizing platelets adhesion (marked with a red arrow) versus spreading (marked with a yellow arrow) on collagen matrix. **B** shows demonstrative images comparing different levels of platelet adhesion and spreading to collagen in one–day stored MM-PCs and CAG-PCs in presence and absence of PP2 treatment. Graph **C** compares number of adhered platelets among MM-PCs, CAG-PCs and PP2-treated CAG-PCs (n = 10) while **D** demonstrates different levels of platelet spreading on collagen matrix in each product during storage. CAG = continuously-agitated; MM = manually-mixed; .PC = platelet concentrate. Note: ns: not significant; *p < 0.05; **p < 0.01

Figure 6A depicts a representative image demonstrating spread one-day stored platelets compared to those that are simply adhered to the collagen matrix, while Fig. 6B shows a comparison between PP2-treated platelets (also under agitation) and untreated controls (including CAG-PCs and MM-PCs). This figure indicated that platelets (one-day stored) treated with 10 μM PP2 lost their ability to spread, whilst remaining adherent to the collagen matrix (Fig. 6B). Examination of platelets during storage also indicated that although treated platelets during 5 days of storage have similar adhesion ability to untreated controls (CAG-PCs and MM-PCs), their spreading capacities were significantly inferior compared to CAG-PCs (Fig. 6 C&D).

### Superior platelet adhesion and spreading over collagen matrix in manually mixed PCs during storage

As shown in representative Fig. 6B, evaluations performed on one-day stored platelets indicate higher levels of adhesion and spreading on collagen in MM platelets compared to those of CAG ones. Figure 6C showed a higher level of platelets adhesion in MM-PCs compared to CAG-ones by the fourth day of storage with significant increases observed in day 1 and day 3 of storage. However, MM-PCs showed a significantly higher spreading over the entire 5-day storage period (Fig. 6D).

### Improved platelet swirling, counts and MPV in manually mixed PCs during storage

**Fig. 7:**
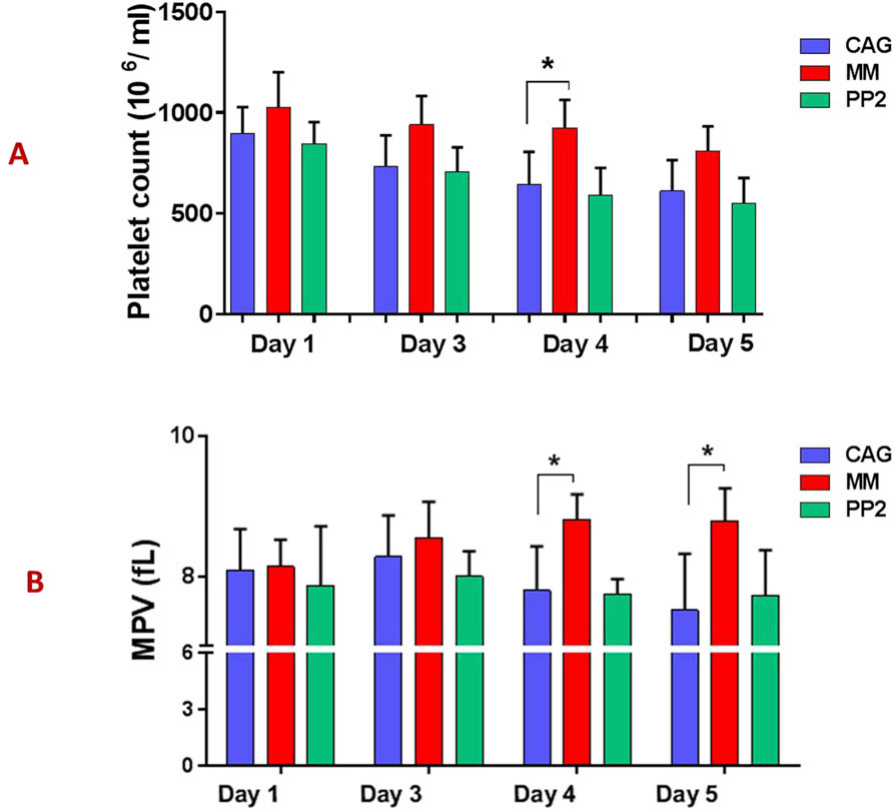
Platelet count and MPV in in manually mixed and agitated PCs during storage. **A** and **B** respectively show the platelet count and MPV in MM-PCs, CAG-PCs and PP2-treated CAG-PCs (n = 10) during storage. MPV = mean platelet volume; CAG = continuously-agitated; MM = manually-mixed; .PC = platelet concentrate. Note: ns: not significant; *p < 0.05

As shown in Fig. 7A & B, MM-PCs showed higher platelet counts and MPV compared to GAG-ones, which was significant on the fourth day of storage for counts and from forth day for MPV. In addition treatment with PP2 did not have a significant effect on platelet count and MPV however as shown in Table 1, PP-2 treated PCs demonstrated inferior swirling compared to other groups while MM-PCs showed better swirling during storage. While all products had acceptable pH levels (according to AABB criteria), there was no significant difference in pH level between either PP2-CAG-PCs or MM-PCs and CAG-PCs during storage.

**Table 1:** Swirling scores and pH levels of PCs under the conditions of manually mixing (MM), continuing agitation (CAG) and CAG with PP2 treatment

	Swirling			pH		
Sample	CAG	MM	PP2	CAG	MM	PP2
Day						
**1**	**3+**	**3+**	**2-3+**	**7.52 ± 0.09**	**7.31 ± 0.06**	**7.41 ± 0.1**
**3**	**2-3+**	**3+**	**1-2+**	**7.59 ± 0.08**	**7.15 ± 0.08**	**7.52 ± 0.07**
**4**	**2+**	**2-3+**	**1+**	**7.41 ± 0.05**	**6.92 ± 0.09**	**7.36 ± 0.08**
**5**	**2+**	**2+**	**1+**	**7.26 ± 0.09**	**6.75 ± 0.06**	**7.19 ± 0.07**

## Discussion

Studies on thrombotic models in pathological conditions indicate the important role of major platelet collagen receptor GPVI in early stages of platelet activation and firm adhesion [25]. Upon reaction with its corresponding ligands, GPVI transduces activating signals which can lead to platelet aggregation, granule release and pro-coagulant function [26, 27]. Platelets lacking GPVI have been shown to not firmly adhere to and aggregate on collagen in vitro [28–30]. As revealed in different experimental thrombosis models, these platelets also failed to build occlusive arterial thrombus in vivo [31]. In addition studying different injury models of carotid artery in vivo, also confirmed the critical role of GPVI in platelet recruitment to the injured arterial wall [32]. From a clinical point of view, several inherited and acquired defects in platelet GPVI have been reported that can result in mild to severe bleeding disorders [33–38]. So far, two alternatively spliced forms and several polymorphisms have been identified for GPVI that are variably associated with changes in platelet function or the risk of thrombosis [39, 40], however GPVI deficiency is thought to be more acquired, usually due to the presence of autoantibodies [38]. This is also confirmed by the fact that the inhibition of platelet GPVI with specific antibodies or antagonizing its binding to immobilized collagen via soluble GPVI dimers reduces arterial thrombosis [41]. Nonetheless regardless of the related mechanisms, GPVI deficiencies appears to cause milder bleeding complications than other adhesion receptors, one of which may be due to compensatory activity of other alternative receptors that provide a relative hemostasis to patients throughout life [42]. Although this adaptive process can be important in clinical cases, the loss of GPVI in stored products or experimental conditions that occurs in a short term may not be readily remedied by the above biological conditions. It is therefore understandable that such platelets may have less clinical efficacy and hemostatic ability, so that their transfusion may not properly manage the bleeding complications in patients due to lower receptors expression or their dysfunction while their loss due to ectodomain shedding can also affects platelet survival in circulation [43, 44]. So far little research is available on GPVI modulation during storage of platelet products. As one of the few studies, Hosseini et al showed an increase in GPVI shedding along with a concomitant decrease in the expression of this receptor in stored platelet concentrates (PCs). In their study, observing an inverse correlation between the expression of platelet activation markers (such as P-selectin) and GPVI, as well as a direct relationship between increasing soluble levels of these markers and GPVI shedding provide suggestions on how to modulate this receptor during PSL. However, the pattern of GPVI shedding for stored platelets differed from what was previously reported in static condition where unlike GPIbα, GPVI does not shed spontaneously and its shedding rate is also slower [45]. Surprisingly, as shown by Hosseini et al in stored platelets under agitation the levels of GPVI shedding was much higher than those expected. One hypothesis was that continuous agitation per se may cause shedding induction, especially since some studies also suggest a role for shear stress in regulating GPVI shedding/expression [15–17]. It should be noted that although such mild agitation may not be considered an important mechanical stress, its long duration in platelet storage may have such a potential effect on platelet structure. In addition, due to the significantly higher concentration of cells in PCs (~ 1 × 10^9^ platelets/mL) compared to the above studies, which in turn increases the collision events, these unique conditions has the potential to produce similar biomechanical effects. In other words, not only may the PSL impose deleterious effects by increasing platelet pre-activity, the continuous collision during agitation may also have an additional effects on lowering GPVI expression; further increasing GPVI shedding/internalization during storage.

However, it is not experimentally straight forward to confirm this theory because parallel comparisons must be made with platelets stored under static conditions, wherein the removal of continuous agitation is inconsistent with product quality [7, 46]. Therefore, static storage conditions could not be used here as a valid comparison model. The observation in this study that manual mixing of PCs showed no adverse effect on platelet quality markers till the 4th day of storage [8] provided us with the opportunity to address the exclusive effect of agitation on platelet activity in a parallel observation. Therefore in this study to address the possible effects of continuous agitation on GPVI modulation, the expression and shedding of this receptor were evaluated under three different conditions of platelet storage: 1. CAG-PCs (continuously agitated platelet concentrates), 2. CAG-PCs in presence of PP2 treatment of platelets (in order to inactivate downstream GPVI signaling pathways in addition to preventing GPVI shedding [43, 47], and 3. MM-PCs (manually mixed PCs) without agitation. Considering PP2 treated platelet as a control that shows the contribution of PSL-dependent activating signals in GPVI modulation, it seems that any significant difference between the levels of GPVI shedding/expression in MM- and CAG-PCs could highlight the exclusive effect of continuous agitation (as the possible factor inducing a biomechanical or shear stress) on the modulation of this receptor, especially in comparative conditions without any evidence indicating different levels of platelet activation between these two groups. Previous studies by Michel et al have examined manual mixing (MM) as an alternative approach for agitation. This study examined some platelet functional activities in PCs stored under manual mixing condition in which they observed no significant changes in metabolic conditions, aggregation responses, release of platelet granular contents (ATP and β-TG) and post-transfusion recovery, compared to continuously agitated platelets. A condition that remained stable for at least four days of platelet storage [8]. Consistent with these reports, we also did not find any inferior metabolic capacities in MM platelets (considering glucose concentration and LDH release- data not shown), while we additionally examined P-selectin expression as an important marker of platelet activation in each group where we showed its insignificant changes in MM platelets compared to those kept under continuous agitation. In addition to previously published data showing comparable aggregation responses to a weak agonist (ADP: 3.2 μM and 32 μM) and a strong agonist (ionophore A23187: 16.7 μM) in manual mixed and agitated PCs [8], the observed comparable P-selectin expression provides further evidence for similar platelet activation state between MM- and CAG-PCs. Given this, we also found superior responses of MM platelets to TRAP compared to agitated-PCs on day one of storage, the observation that may attribute a good quality score to MM-PCs [48]. However, PP2-treated PCs showed a significant reduction in P-selectin expression, an observation consistent with the findings of other researchers in vitro [49] and may indicate successful inhibition of platelet activity in this condition. In this study, compared to CAG-PCs, PP2 treated PCs under agitation have been initially used as control platelets with inhibited signaling activity in order to understand the unique effects of agitation on platelet GPVI modulation and adhesion capacities on collagen. In the case of GPVI, a specific reason for using PP2 could be its successful inhibitory effect on the shedding and downstream signaling of this receptor, as several studies have shown the important role of Src kinases in the regulation of both ADAMs-dependent shedding and ITAM-mediated signaling of this receptor [43, 47]. Consistently we also found a significant reduction in GPVI ectodomain shedding in PP2 treated platelets during storage which was consistently associated with higher levels of GPVI expression compared to platelets conventionally stored under CAG. A decrease in shedding and corresponding increase in receptor expression in PP2-treated platelets may be expected due to attenuated platelet signaling capacities, however the observations of much higher GPVI expression in MM platelets while they had GPVI shedding comparable to CAG platelets attracted our main attention and interest.

Actually normal platelet has a dynamic state with a balance between activation-dependent increases in surface expression of receptors versus their gradual loss due to ectodomain shedding, internalization or microvesiculation, all of which are also caused by platelet activation [44]. Specifically for GPVI, in addition to surface expression, the receptor is also present on open canalicular system and α granules, pools that are not expressed by resting platelets, whereas after stimulation they merge with the plasma membrane content while increasing GPVI expression by approximately 60% [50]. However, it should be noted that stimulation of platelets with high concentration of potent agonists can prevent an increase in surface levels of GPVI and even reduce receptor expression by inducing severe ectodomain shedding of receptor [51, 52]. Similarly here, in stored PCs that experienced a gradual increase of platelet activation, net GPVI expression is defined by the ultimate balance between gradual increment in receptor and its loss due to shedding or internalization events. We simulated this condition in an experiment in which we showed that using 1uM ionophore, platelets experienced a significant increase in GPVI expression, whereas by stimulating 10 μM ionophore, hyperactive platelets severely lost their GPVI expression, to the extent that it is even much lower than its initial threshold. In other words, in the latter case, the platelet activation levels are very high, which leads to the dominance of GPVI shedding over its increased expression. This prevents GPVI restoration on platelet surface, the phenomenon that may also occur during platelet storage. These mechanisms are attributed to normal platelets, whereas in PP2-treated platelets, inhibition of Src kinase partially prevents platelet activation during storage. This means that while these platelets exhibit significantly reduced shedding, they are also experiencing an inhibition of gradual increase in GPVI expression during storage. This can also be simulated in an experiment in which an agonist is added to platelets in the presence of an inhibitor, resulting in no significant increase in agonist-induced activation or receptor expression. Here either MM or CAG platelets have their dynamic functions with both activation-dependent loss of GPVI (due to shedding, internalization or microvesiculation) and increasing levels of GPVI expression together during storage,whereas in PP-2 treated platelets the activation pathways are partially blocked. Therefore, according to shedding/expression ratios in three different conditions of MM, CAG and PP2 treated CAG platelets, obviously MM-PCs are better achieved to keep or compensate their GPVI expression regardless of higher levels of shedding than PP-2 treated PCs with a constant ratio of shedding and expression. However comparing to CAG-PCs with the comparable levels of GPVI shedding, significantly higher expression of these receptors in MM platelets can be due to other possible down-regulation mechanisms including biomechanical loss of receptors, internalization or microvesiculation which may be enhanced by agitation. Here regardless of higher GPVI levels in PP2-treated CAG platelets compared to CAG ones, which might be due to partial inhibition of shedding in presence of Src kinase inhibitor (reduced ADAMs activity), the significant reducing trend of GPVI expression in treated samples, may also be due to those agitation-dependent losses of GPVI which even occurs in presence of PP2.

On the other hand, considering the similar levels of platelet activity between MM and CAG-PCs, the issue of no significant difference in the levels of GPVI shedding in these two groups can be logically interpreted. However, why receptor expression levels are higher in MM products without evidence of increased platelet activity is an important question, especially since in comparison; the ratio of GPVI shedding to its expression in MM-PCs was also much lower than those of CAG-PCs. Therefore, regardless of the mechanism behind the increased expression of GPVI in MM-PCs, the receptor shedding ratio here indicates that lack of continuous agitation has been able to effectively reduce the trend of relative GPVI shedding compared to platelets under continuous agitation. In other words, these observations, together with the findings regarding higher GPVI expression in PP2-CAG-PCs, suggest that the lower GPVI expression in CAG-PCs may be also due to the mechanical challenge imposed by agitation, which reduces GPVI receptor expression, now either by increasing the shedding ratio or by internalizing the receptor [44].

The role of Mechanical stress in in inducing of GPVI shedding has also been proposed by several other studies [15–17]**.** More interestingly, from a functional point of view, these differences in receptor expression and shedding were similarly emerged in platelet adhesion studies, to the extent that both platelet adhesion and spreading on collagen were higher in MM products than in GAG ones. These findings are consistent with our previous studies, which suggested a direct correlation between GPVI modulation and platelet adhesion capacity to collagen during storage [19, 53]. However, the interpretation of these studies is complicated by the additional contribution of storage-dependent metabolic stress, which may have overshadowed the potential effect of reduced GPVI expression on platelet adhesion deficiency. With the final interpretation that the main reason for the decrease in adhesion capacity in PCs that are stored for a longer period of time, could be due to metabolic stress rather than reduced GPVI expression. Remarkably, another advantage of the present study is that under equal metabolic conditions, MM- platelets with higher adhesion/spreading rates than CAG-ones also had much higher GPVI levels at the same time. Therefore, given the similar metabolic conditions here for each pair product, a direct relationship between GPVI expression and spreading can be easily interpreted, which could confirm the importance of this receptor in ensuring proper platelet spreading on the collagen matrix during storage. This observation is in line with a study by Chen et al., in which they showed GPVI-mediated collagen responses are relevant to surface GPVI expression of platelets in a receptor density-dependent fashion [54]. However for PP2-platelets regardless of higher expression of GPVI, they did not show any enhanced adhesion capacities compared to platelets obtained from GAG-PCs. In addition, PP2-platelets showed even impaired spreading on collagen. This finding highlights the fact that inhibition of Src kinase, while not affecting platelet adhesion, prevents the ability of platelets to spread on collagen [55–57]. Similarly here, our functional flow cytometric study also showed the lack of increased expression of GPVI in response to collagen in PP2-treated platelets compared with untreated platelets, the observation that confirms the aforementioned findings of platelet adhesion on collagen matrix. This is another observation that may also support the importance of GPVI down-stream signaling for proper spreading of platelets on collagen.

Another interesting finding of this study was the evaluation of some common qualitative markers of platelets such as pH, swirling and platelet count during storage. Our comparison between MM and CAG-PCs does not show a significant difference in pH, which further confirms the similarity of the metabolic conditions of these products. However, swirling (especially at the forth days of storage) were significantly improved in manual mixing condition, a finding that highlights previous studies suggesting a possible negative impact of agitation-mediated shear stress on quality indices in PCs [1, 12–14]. Conversely, in PP2-CAG-PCs, platelets clearly showed lower swirling, and this observation, along with their impaired spreading capacities on collagen may highlight the importance of Src-kinases threshold activity in maintaining platelets quality during storage. More intriguingly lower MPV and reduced platelet count in CAG-PCs may also be due to agitation-dependent membrane microvesiculation because, as previously shown, microvesiculation is one of the main factors in reducing these two platelet indices. However, whether agitation-dependent biomechanical forces per se can induce higher levels of platelet microvesiculation, and whether this possible event affects GPVI expression [44], requires further study.

## Conclusion

There are several lines of evidence that the activation state of platelets in daily manually-mixed PCs can be comparable to that in PCs under normal continuous agitation for up to 4 days. While these findings suggest manual mixing as an alternative technique to maintain platelet quality and efficacy under certain conditions (such as lacking access to a suitable appropriate agitator), our observations of increased platelet adhesion/spreading, higher GPVI expression, and better swirling under this condition, may be somehow attributed to down-regulation of platelet functional capacity in response to agitation. These observations underscore the importance of previous studies that mechanical shear stress can modulate GPVI-dependent function due to increased ectodomain shedding of this receptor. While this is the main message of study presented here, we emphasize to draw readers’ attention to the fact that our goal is not to recommend manual mixing as a usual alternative to agitation, unless there is no access to agitator, which is only valid for a limited period of 4 days.

## Supplementary Information

**Additional file 1.**

## Data Availability

The corresponding author can make available some dataset upon reasonable request.
