# Correction to: agitation-dependent biomechanical forces modulate GPVI receptor expression and platelet adhesion capacity during storage

**DOI:** 10.1186/s12959-022-00371-5

**Published:** 2022-04-06

**Authors:** Ehteramolsadat Hosseini, Amin Solouki, Masood Haghshenas, Mehran Ghasemzadeh, Simone M. Schoenwaelder

**Affiliations:** 1 Blood Transfusion Research Centre, High Institute for Research and Education in Transfusion Medicine, Tehran, Iran; 2 Charles Perkins Centre, The University of Sydney, Camperdown, NSW Australia; 3 Heart Research Institute, Newtown, NSW Australia

**Correction to:** ***Thrombosis J*** **20, 3 (2022)**.

**https://doi.org/10.1186/s12959-021-00359-7**

Following publication of the original article [1], it was reported that Fig. 2, Fig. 6 and Table 1 contained several typographical errors. A table header and a label in both figures were incorrectly labelled ‘MAN’ instead of ‘MM’.

**Fig. 2:**
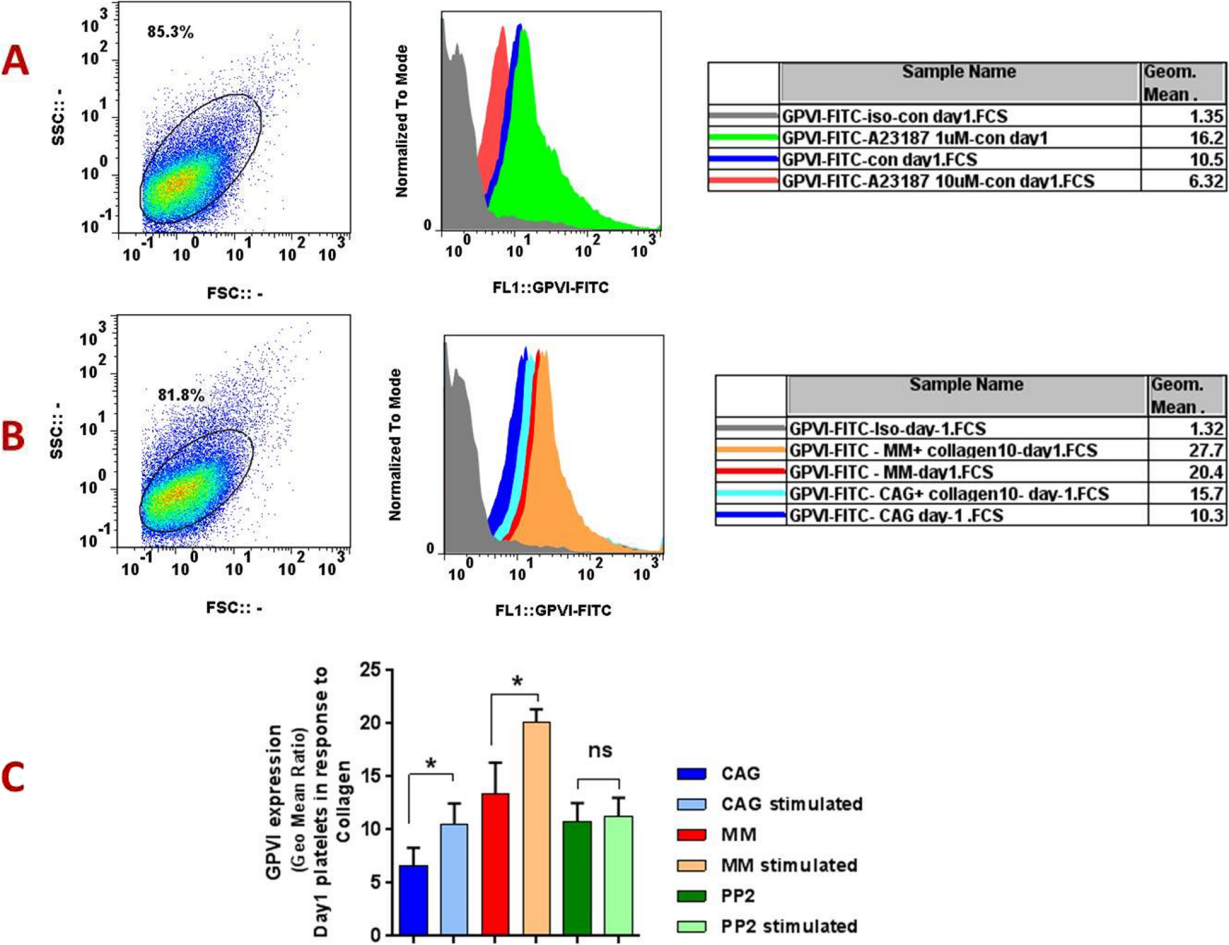
GPVI expression in response to different agonists. The representative histograms show different levels of GPVI expression in response to low (1 μM) and high (10 μM) concentrations of potent agonist ionophore A23187 (A) as well as 10 μg/ml collagen (B) in one-day stored CAG platelets. Graph (C) also depicts the significant increments of GPVI in response to collagen in both MM- and GAC- PCs whereas this agonist did not increase GPVI expression in PP2 treated GAG-PCs (all in one-day stored PCs, *n* = 5). CAG = continuously-agitated; MM = manually-mixed;.PC = platelet concentrate. Note: ns: not significant; *p < 0.05

**Fig. 6:**
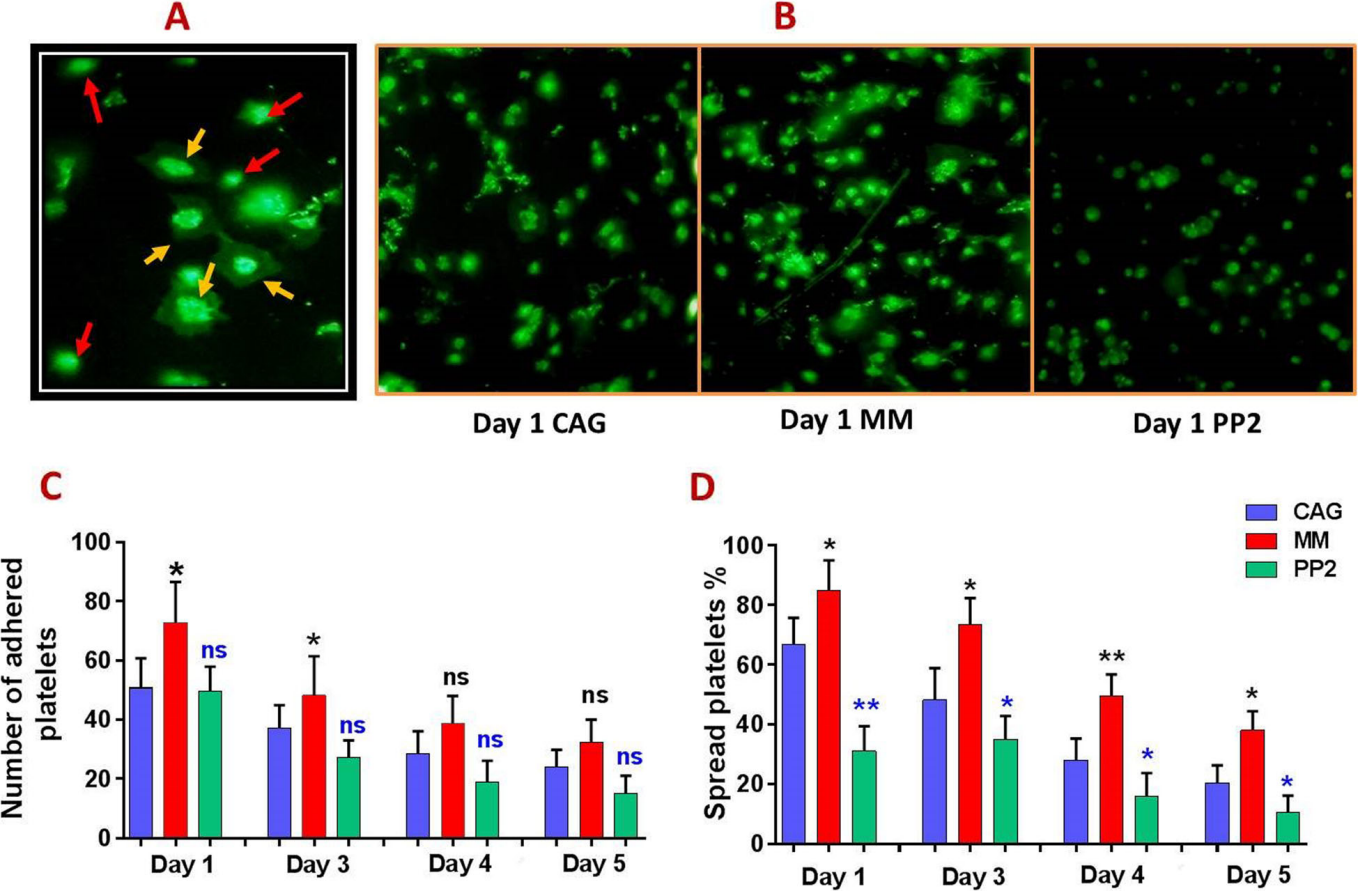
Platelet adhesion and spreading over collagen matrix in manually mixed and agitated PCs during storage. A provides a demonstrative image characterizing platelets adhesion (marked with a red arrow) versus spreading (marked with a yellow arrow) on collagen matrix. B shows demonstrative images comparing different levels of platelet adhesion and spreading to collagen in one–day stored MM-PCs and CAG-PCs in presence and absence of PP2 treatment. Graph C compares number of adhered platelets among MM-PCs, CAG-PCs and PP2-treated CAG-PCs (*n* = 10) while D demonstrates different levels of platelet spreading on collagen matrix in each product during storage. CAG = continuouslyagitated; MM = manually-mixed; .PC = platelet concentrate. Note: ns: not significant; *p < 0.05; **p < 0.01

**Table 1:** Swirling scores and pH levels of PCs under the conditions of manually mixing (MM), continuing agitation (CAG) and CAG with PP2 treatment

	Swirling			pH		
Sample	CAG	MM	PP2	CAG	MM	PP2
Day						
**1**	**3+**	**3+**	**2–3+**	**7.52 ± 0.09**	**7.31 ± 0.06**	**7.41 ± 0.1**
**3**	**2–3+**	**3+**	**1–2+**	**7.59 ± 0.08**	**7.15 ± 0.08**	**7.52 ± 0.07**
**4**	**2+**	**2–3+**	**1+**	**7.41 ± 0.05**	**6.92 ± 0.09**	**7.36 ± 0.08**
**5**	**2+**	**2+**	**1+**	**7.26 ± 0.09**	**6.75 ± 0.06**	**7.19 ± 0.07**

The corrected figures and table are included in this Correction article and the original article [1] has been updated.
