# Supplementary material for: Agitation-dependent biomechanical forces modulate GPVI receptor expression and platelet adhesion capacity during storage
Source: Thromb J. 2022 Jan 12;20:3. doi: 10.1186/s12959-021-00359-7 (PMC8756730; doi:10.1186/s12959-021-00359-7)
Supplement: Supplementary file 1 — Additional file 1. [file 12959_2021_359_MOESM1_ESM.docx]

**Supplementary Method and Materials**

**Reagents**

Mouse IgG1 κ Isotype controls (PE or FITC conjugated) were from Miltenyi Biotec (Germany). Monoclonal antibodies against Human GPVI (PE conjugated; clone: HY101) and CD61 (PE conjugated; clone: VI-PL2) were from eBiosciences Inc (USA), while other FITC mouse anti human GPVI (clone: H-5) was also purchased from Santa Cruz Biotechnology. CD62p (P-selectin; FITC and PE conjugated; clone: AC1.2) were from BD Pharmingen (USA). For western blot analysis, Monoclonal antibodies against human GPVI (clone HY101) as well as anti-mouse IgG1 HRP were from eBiosciences Inc (USA). Polyclonal anti GPVI (clone: H-139) were also purchased from Santa Cruz Biotechnology (USA). Other reagents and chemicals were from Sigma Aldrich (USA).

**Sample preparation:**

20 PRP-PCs units obtained from the eligible, volunteer donors after informed consent. All products passed release process under Iranian Blood Transfusion Organization (IBTO) standard protocols. Each bag contained at least 1×10^9^ platelets/mL in ~70 mL of autologous plasma (based on AABB criteria). Each two ABO- and D- matched PRP-PC units were pooled in a closed system using a connecting device instrument (TSCD-II, Terumo Sterile Tubing Welder, Japan). The obtained new bag was mixed manually for a while and again subjected to connection device to be splitted into three new platelet bags from same company, while adjusting same volumes (still met the standard limit) for each with the used a digital scale. The bags labeled as GAC-PC (PC kept under continuous agitation), PP2-CAG-PC (CAG-PC treated with Src kinase inhibitor PP2) and MM-PC (PC stored with manual mixing without agitation according to described method by Mitchell et al [1]. Then under sterile condition, designated amounts of each PP2 inhibitor solved in the buffer were added to the indicated bags (to provide the final concentration of 10μM) while the same buffer without inhibitor (sham) was also added to either GAC-PC or MM-PC. All bags were then kept at 20–24 °C in designated agitation condition until the time of sample preparation when under sterile conditions; 2 mL of products was taken from cord for the required analysis on days 1, 3, 4 and 5 of storage. For each part of study washed platelets were isolated and re-suspended in Tyrode’s buffer as described previously [2] . For flow cytometry and adhesion analysis, platelet count was adjusted to 2×10^7^ /ml. Platelet-poor plasma (PPP), obtained from PRP with the platelet count of 5×10^8^ /mL were also subjected to two steps ultracentrifugation (2×10^4^g for 30 min each time) and MP-free supernatants were separated and kept in –20 °C to be analyzed by western blot (WB) or ELISA [3] . The study was approved by the local ethical committee, and the informed consent was obtained from the blood donors by IBTO.

**Flow cytometry analysis to determine the levels of surface adhesion molecule GPVI and P-selectin expression**

Platelets were stained with either fluorochrome-conjugated anti CD61, anti CD62P (P-selectin) or anti-GPVI for 30 min. For each receptor expression, isotype matched control antibody was used to determine background staining created by non-specific binding. Cells were then fixed in 1% paraformaldehyde in PBS and subjected to flow cytometer (CyFlowSpace, Partec GmbH, Germany) where a total of 20000 platelet events were acquired. The flow cytometer settings were optimized for the acquisition of platelets by logarithmic signal amplification in all four detectors (forward and side scatter channels and fluorescence channels FL1 and FL2). For analysis, the gate was set around intact platelet population as defined by forward and side scatter characteristics and confirmed by the presence of platelets expressing CD61. The percentage of positive platelets expressing P-selectin above the background (negative control) was recorded while GPVI expression was quantified as the mean fluorescence intensity (MFI). Notably, in this study MFI was presented as the geometric mean (Geo Mean) which is obtained by mathematical calculation to evaluate Mean Fluorescent Intensity on a logarithmic scale. On the other word Geo Mean is an arithmetic average of MFI on a log scale. Data were analyzed with FLOWJO software (Tree Star Inc., OR, USA) and the ratio of Geo Mean is calculated with dividing the Geo Mean of the samples by the amount of isotype control.

**Measurement of GPVI plasma level by ELISA (semi-quantitative method)**

In brief, using ELISA plate, wells were coated with monoclonal mouse anti-human GPVI antibody at room temperature. Wells were washed with PBS containing 0.05% Tween 20 .the wells were then blocked with 1% BSA at room temperature. As positive controls, dilutions of ultra-centrifuged plasma obtained from fresh PRP treated with ionophore (2 μM) were prepared. Then 100 μl of positive controls as well as the samples (Microparticle-free PPP, obtained from PRP with the platelet count of 5×10^8^ /mL) were added to each well and incubated at room temperature with a gentle shake. Followed by incubation and washing steps, 100 μl of biotinylated anti-human GPVI-polyclonal was added to each well and incubated again at room temperature with a gentle shake. Washing was repeated and in the next step, 100 μl of HRP streptavidin solution was added to each well. Again, following the incubation period and washing steps, 100 μl of single-stage TMB substrate was added to each well. The plate was kept at room temperature in a dark place where after an additional incubation time, 50 μl of stop solution (two normal H2SO4) was added to each well and the light absorption (optical density, O.D) was immediately read at a wavelength of 450 nm by the ELISA-reader. The average absorbance for each controls and samples were recorded. The absorbance of the controls with serial dilutions was linear which indicated the optimal performance of the experiment. To evaluate the semi-quantitative values of the samples, the ratio of each sample absorbance to the light absorption obtained from control (2 μM ionophore- treated PRP) was calculated.

**Western blotting to analyze GPVI shedding**

To validate our ELISA method for sGPVI analysis, some Microparticle-free Platelet-poor plasma (PPP), obtained from PRP with the platelet count of 5×10^8^ /mL were subjected to SDS-PAGE and western blotting analysis. To prevent interfere with the proteins bands, albumin was fractionated from samples before experiments [4]. Proteins were then Immunoblotted using primary specific monoclonal antibody, HRP-conjugated secondary antibody and enhanced chemiluminescence reagent. Finally, bands were visualized by ChemiDoc XRS+ system using image Lab software (Bio-Rad Laboratories, inc. USA). For each run of shedding analysis, the free-MPs supernatant of samples obtained from PRP-PCs (with the same count of platelets) were subjected to western blot analysis along with a positive control of shedding. For each run, the intensities of samples were calculated in order to [semi-quantitative](https://www.google.com/url?sa=t&rct=j&q=&esrc=s&source=web&cd=1&cad=rja&uact=8&ved=0CBwQFjAAahUKEwiUitu7kZfJAhXDvhQKHcaABXc&url=http%3A%2F%2Fwww.merriam-webster.com%2Fdictionary%2Fsemiquantitative&usg=AFQjCNFUOTYtf7jD_QHzmaUhy46VDCXkvw&bvm=bv.107467506,d.bGg)ly analyze and compare the amounts of shed receptors from day 1 to day 5 of storage.

**Static platelet adhesion to collagen matrix**

Glass coverslips (12 mm in diameter) were incubated with 100 μg/ml collagen type I in PBS for 1hr at room temperature and then followed by washing steps, those coverslips incubated again with 2% bovine serum albumin (as blocking solution) for 30 minutes at room temperature. Excess solution was removed by three washes with Tyrode’s buffer and coverslips were kept immersed in Tyrode’s buffer until required. Human platelets (2×10^7^/mL) were then allowed to adhere on coverslips for 30 min at 37 °C under low stirring condition. Non-adherent platelets were aspirated and adherent platelets fixed with 3.7% formaldehyde for 15 min. Adherent platelets were visualized by fluorescence microscope (100x objectives). For this purpose prior to each experiment platelet were labeled with fluorescence dye, DIOC6 and then platelets were subjected to adhesion assays. Total number of adhered platelets and of those the percentages of spread platelets were calculated.

**QC parameters of PCs**

PH evaluation was performed at 22 ° C on pH meters (826 pH mobile / 827 pH lab, Metrohm AG, Switzerland) at each PC storage time. Notably both MM- and CAG- PCs had comparable pH in average standard limit within 4 days of storage whereas for MM- PCs pH values were lower after 5 days of storage. PLT count and MPV were measured using a hematology full blood analyzer (XE-2100, Sysmex, Milton Keynes, UK). ***Bacterial cultures*:** According to the QC protocol, to confirm that PCs were not contaminated upon receipt or did not get contaminated during storage; bacterial cultures (including both aerobic and anaerobic cultures) were performed on the samples obtained from each bags of PCs during first and last days of their storage. Fortunately, we did not detect any bacterial contamination in our used products

**Statistical analysis**

*Mann*–*Whitney U test* was applied to compare parameters between each two groups (either agitated PCs treated with inhibitor or manually mixed PCs compared to control agitated PCs). P values of less than 0.05 were considered to be significant. Analyses were performed by GraphPad Prism software (GraphPad Prism Software, Inc., San Diego, CA).

**References**

1. Mitchell S, Hawker R, Turner V, et al. Effect of agitation on the quality of platelet concentrates. Vox sanguinis 1994;67:160-165.

2. Kulkarni S, Woollard KJ, Thomas S, et al. Conversion of platelets from a proaggregatory to a proinflammatory adhesive phenotype: role of PAF in spatially regulating neutrophil adhesion and spreading. Blood 2007;110:1879-1886.

3. Ghasemzadeh M, Hosseini E, Roudsari ZO, et al. Intraplatelet reactive oxygen species (ROS) correlate with the shedding of adhesive receptors, microvesiculation and platelet adhesion to collagen during storage: does endogenous ROS generation downregulate platelet adhesive function? Thrombosis research 2018;163:153-161.

4. Fu Q, Garnham CP, Elliott ST, et al. A robust, streamlined, and reproducible method for proteomic analysis of serum by delipidation, albumin and IgG depletion, and two-dimensional gel electrophoresis. Proteomics 2005;5:2656-2664.
